# Acceptance of midwifery qualification acquisition by gender identity and sexual orientation: a survey of nursing students

**DOI:** 10.20407/fmj.2025-024

**Published:** 2026-02-28

**Authors:** Keisuke Iwase, Hironori Tsuzuki, Mikiko Shimizu

**Affiliations:** 1 Faculty of Nursing, Fujita Health University, School of Health Sciences, Toyoake, Aichi, Japan; 2 School of Health Science, Department of Nursing, Toyohashi Sozo University, Toyohashi, Aichi, Japan

**Keywords:** Midwifery license, Sex-based restriction, Gender identity, Nursing students, Social acceptance

## Abstract

**Objective:**

Amid growing calls for respect for gender diversity, Japanese law restricts midwifery qualifications to women, excluding individuals assigned male at birth. By contrast, in some countries, all individuals can pursue midwifery qualifications regardless of gender. These differences highlight varying institutional frameworks and levels of social acceptance. To clarify nursing students’ acceptance of midwifery qualification acquisition across gender identities and sexual orientations.

**Methods:**

An anonymous online survey was conducted from April to May 2024 among 98 undergraduate nursing students (10 men and 88 women) enrolled at a single anonymous health sciences university in Japan. Acceptance of midwifery qualification acquisition was assessed using the proportion of positive evaluations for nine gender identity and sexual orientation categories. Evaluations were measured on a 4-point Likert scale. Data were analyzed using descriptive statistics, cross-tabulation, and χ^2^ tests.

**Results:**

Heterosexual women had the highest level of acceptance (96.9% positive evaluations), while heterosexual and bisexual men had the lowest (46.9%). There were substantial gender differences in the level of acceptance for different sexual orientations; however, none were statistically significant.

**Conclusion:**

Acceptance of midwifery qualification acquisition varied by gender identity and sexual orientation categories, suggesting that gender roles and cultural perspectives may influence attitudes. The findings indicate that systems should be reviewed to allow anyone, regardless of gender, to qualify and work as a midwife. They also highlight the importance of fostering an understanding of diversity through education.

## Introduction

In recent years, discussions and legal reforms regarding diverse gender identities, including LGBTQ+ identities, have progressed worldwide. In Japan, approximately 9.7% of the population identifies as LGBTQ+.^1^ A 2023 Supreme Court ruling found that the requirements for legal gender change were unconstitutional,^2^ highlighting the growing need for institutional reforms recognizing gender diversity.

While nursing licenses in Japan can be obtained regardless of gender, the Act on Public Health Nurses, Midwives, and Nurses^3^ restricts midwifery licenses to women. Individuals assigned male at birth are not eligible to obtain this license. However, many countries, such as the United States and the United Kingdom, allow midwifery qualifications to be obtained regardless of gender identity or sexual orientation.^4^

Against the backdrop of declining birth rates, the growing need for infertility treatments, and increasing maternal age, the role of midwives has become even more crucial. Recruiting midwifery professionals from diverse groups is therefore a pressing concern. However, in Japan, institutional restrictions and social prejudices related to gender roles may impose psychological and structural barriers for aspiring midwives. In international practice, male and transgender midwives have reported facing isolation and institutional challenges in educational and clinical settings.^5^ Thus, as well as expanding individuals’ formal eligibility to obtain these qualifications, social acceptance and support systems must also be developed.

A cross-sectional survey of nursing students in the Philippines revealed that many students expressed positive attitudes toward LGBT individuals. However, their level of knowledge regarding LGBT identities was moderate, and no significant correlation was found between their levels of knowledge and attitudes.^6^ Furthermore, research focusing on attitudes toward the acceptance of midwifery qualifications remains extremely limited, both domestically and internationally.

This study aimed to clarify nursing students’ acceptance of midwifery qualification acquisition across nine different gender identity and sexual orientation categories, and to provide foundational data for future educational programs and institutional improvements.

## Methods

### Study design

A cross-sectional descriptive survey.

### Participants and data collection

An anonymous online survey was conducted using Google Forms from April to May 2024 among undergraduate nursing students (Years 1–4) enrolled at a single anonymous health sciences university in Japan. Recruitment took place through institutional emails and in-person presentations. Students who first enrolled in 2020 were excluded due to a lack of comparability.

### Survey items

There were three survey items: (1) gender, (2) year of enrollment, and (3) level of acceptance of midwifery qualification acquisition. Acceptance was assessed for each gender identity and sexual orientation combination. Nine combinations were selected based on previous studies of gender identity and sexual orientation^1^ and definitions from the Human Rights Campaign^7^ and GLSEN.^8^

1. Women (heterosexual)
2. Men (heterosexual)
3. Women (homosexual/lesbian)
4. Men (homosexual/gay)
5. Women (bisexual)
6. Men (bisexual)
7. Transgender women (assigned male at birth; female identity)
8. Transgender men (assigned female at birth; male identity)
9. Questioning (uncertain gender identity or sexual orientation)

To reduce participant burden, the minimum number of items necessary to address the research objective were included. Although additional questions about age and recognition of the role of the midwifery association were asked, only items directly related to the main objective were analyzed and reported.

### Data analysis

The level of acceptance was measured using a 4-point Likert scale (1=very unfavorable, 4=very favorable). Frequencies, proportions, and means (±standard deviations) were calculated for each category, and the distribution of responses was visualized using bar graphs. Scores of 3 or 4 were considered to be positive evaluations, and scores of 1 or 2 were considered to be negative evaluations. Proportions of positive responses were then calculated for each category. Associations between these proportions and gender and year of enrollment, respectively, were examined using cross-tabulations and χ^2^ or Fisher’s exact tests. Statistical significance was set at a two-sided p-value <0.05. Data analysis was performed using SPSS Statistics (version 27; IBM Corp.).

### Ethical Considerations

This study was approved by the Ethics Committee of Fujita Health University (HM24-270).

## Result

### Participant background

Of 562 nursing students invited to participate, 108 responded (response rate: 19.2%). After excluding 9 students who declined participation and 1 student admitted in 2020, data from 98 students (10 men, 88 women) were analyzed (valid response rate: 17.4%).

### Distribution of acceptance levels of midwifery qualification by gender identity and sexual orientation

**Figure 1:**
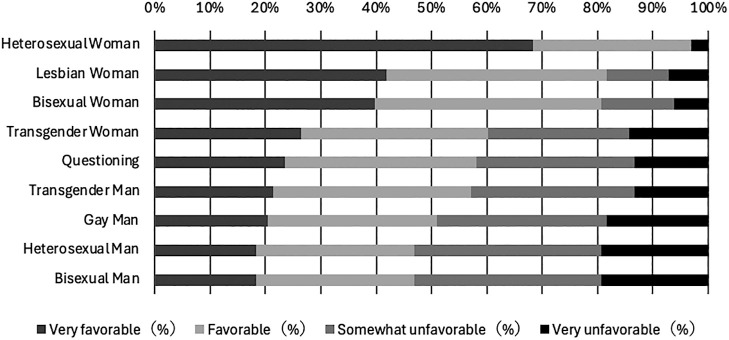
Distribution of evaluations for acceptance of midwifery qualifications across gender identity and sexual orientation categories Note: Categories are displayed in descending order of the proportion of positive evaluations (“Favorable” and “Very favorable”).

Heterosexual women had the highest level of acceptance (96.9% positive evaluations), followed by homosexual women (81.6%) and bisexual women (80.6%; Table 1). The lowest levels of acceptance were reported for heterosexual and bisexual men (both 46.9%; Table 1). For the other categories, positive evaluation proportions exceeded 50.0% (Table 1), including transgender women (60.2%), questioning individuals (58.2%), transgender men (57.1%), and homosexual men (51.0%). Overall, categories incorporating a female gender identity tended to receive more positive evaluations, whereas those with a male gender identity tended to receive less (Figure 1).

### Association with respondents’ gender

Fisher’s exact test showed no statistically significant differences between genders (p>0.05; Table 2). Although not statistically significant, gender differences of 10–30 percentage points were observed. The proportions of positive evaluations differed between genders (Table 2): 70.0% of men provided positive evaluations for transgender women, compared to 59.0% of women (p=0.735); and 20.0% of men provided positive evaluations for heterosexual men compared to 50.0% of women (p=0.098).

### Association with year of enrollment

The distribution by year of enrollment was as follows: 24 students enrolled in 2021 (17.6%), 25 in 2022 (19.1%), 26 in 2023 (17.3%), and 23 in 2024 (15.9%). The supplementary analysis showed higher positive evaluations among students enrolled in 2022 and 2023. Significant differences were observed for evaluations of heterosexual men and bisexual men(p<0.05; Supplementary Material 1).

## Discussion

This novel study clarifies nursing students’ acceptance of midwifery qualifications for different combinations of gender identities and sexual orientations. Only two categories—heterosexual and bisexual men—had positive evaluation proportions below 50%. The remaining seven categories received primarily positive evaluations. This suggests that evaluations were not solely based on physical sex (biological sex). Instead, they may have been influenced by cultural images of midwives and expectations regarding gender roles. In addition, gender differences of 10–30 percentage points were observed in the proportion of positive evaluations for transgender women and heterosexual men. This indicates that psychological proximity toward same-gender categories and differing perceptions of gender roles may have influenced these evaluations. Future studies should verify gender-specific evaluation trends through large-scale quantitative research and further explore the underlying factors through qualitative studies.

Significant differences were observed in the proportion of positive evaluations of heterosexual and bisexual men by year of enrollment. Students who first enrolled in 2022 and 2023 provided more positive evaluations. However, given the limited number of respondents, these results may have been affected by selection or detection bias. There is insufficient evidence to directly attribute them to educational environments or social contexts. Therefore, generalization is difficult, and larger-scale studies are needed in the future.

The history of midwifery in Japan dates back to the Edo period and midwifery was institutionalized during the Meiji era.^9^ Previous studies have shown that women tend to prefer being assisted by other women during childbirth, reflecting a strong cultural expectation.^10^ In this context, the perception of midwives as “maternal women who support childbirth” remains prevalent. Furthermore, this perception may be reinforced by the fact that men in Japan are legally prohibited from obtaining midwifery qualifications.^3^ Such entrenched values and institutional restrictions may shape social recognition and unconsciously influence career choices and evaluations. Studies on transgender and gender-expansive healthcare professionals have reported experiences of psychological distress due to transphobia, feelings of isolation resulting from the gender binary, and institutional barriers, underscoring the importance of developing supportive environments.^11^

In this study, categories that were more aligned with the cultural image of midwives tended to be evaluated positively, whereas those that were less aligned were more likely to receive negative evaluations. Perceptions of midwives are not only influenced by gender, but also by social and economic backgrounds and personal experiences.^12^ Nursing students are considered more likely to show an understanding of gender diversity; however, previous studies have reported unconscious biases against sexual minorities among healthcare professionals.^13^ Neutral attitudes^14^ and insufficient knowledge among nursing students^15^ must also be considered. While this study examined the acceptance of midwifery qualification acquisition, the social acceptance of actual midwifery practice may differ. Future research should compare the acceptance of qualification acquisition with that of service provision.

The findings of this study can be applied to the development of teaching materials and training programs that present diverse images of midwives and reduce prejudice based on gender and sexual orientation in nursing education. Moreover, the results are consistent with the international trend in which countries are abolishing gender-based requirements. They provide important implications for revising gender restrictions on midwifery qualifications and designing systems that allow the participation of diverse human resources.

### Limitations

The small sample size of 98 participants may have resulted in an insufficient statistical power for comparisons across categories. The study population was restricted to nursing students, and their attitudes may differ from those of the general public. As the survey was conducted online, the responses may have been influenced by social desirability bias or respondent characteristics. Considering these limitations, future studies should involve larger-scale surveys with more diverse participants.

## Conclusion

This study aimed to clarify the social acceptance of midwifery qualification acquisition by gender identity and sexual orientation. It examined nursing students’ attitudes toward nine combinations of gender identity and sexual orientation. The results showed that women tended to receive higher positive evaluations, whereas men tended to receive lower evaluations. At the same time, categories with non-heterosexual orientations or genders that differed from the sex assigned at birth also received positive evaluations, suggesting broader social acceptance. Future research should compare evaluation trends across different backgrounds and accumulate evidence that can contribute to the development of educational programs and institutional reforms.

## Figures and Tables

**Table 1:** Frequency and descriptive statistics of acceptance of midwifery qualifications across gender identity and sexual orientation categories

Category	Very favorable [n(%)]	Favorable [n(%)]	Somewhat unfavorable [n(%)]	Very unfavorable [n(%)]	Average score±SD	Positive [n(%)]	Negative [n(%)]	Total (n)
Heterosexual Woman	67(68.4)	28(28.6)	0(0.0)	3(3.1)	3.62±0.65	95(96.9)	3(3.1)	98
Lesbian Woman	41(41.8)	39(39.8)	11(11.2)	7(7.1)	3.16±0.89	80(81.6)	18(18.4)	98
Bisexual Woman	39(39.8)	40(40.8)	13(13.3)	6(6.1)	3.14±0.87	79(80.6)	19(19.4)	98
Transgender Woman	26(26.5)	33(33.7)	25(25.5)	14(14.3)	2.72±1.01	59(60.2)	49(39.8)	98
Questioning	23(23.5)	34(34.7)	28(28.6)	13(13.3)	2.68±0.97	57(58.2)	41(41.8)	98
Transgender Man	21(21.4)	35(35.7)	29(29.6)	13(13.3)	2.65±0.96	56(57.1)	42(42.9)	98
Gay Man	20(20.4)	30(30.6)	30(30.6)	18(18.4)	2.53±1.01	50(51.0)	48(49.0)	98
Heterosexual Man	18(18.4)	28(28.6)	33(33.7)	19(19.4)	2.46±1.00	46(46.9)	52(53.1)	98
Bisexual Man	18(18.4)	28(28.6)	33(33.7)	19(19.4)	2.46±1.00	46(46.9)	52(53.1)	98

Note: Responses were measured on a 4-point Likert scale (1=very unfavorable to 4=very favorable). For each category, the proportion of responses (%) for each option are presented, as well as the mean score and standard deviation (Mean±SD) for each category. Scores of 1 or 2 were categorized as “Negative” and scores of 3 or 4 as “Positive,” with the number of responses (n) and percentages (%) shown for each. Percentages may not sum up to 100% because of rounding.

**Table 2:** Proportions of positive evaluations across gender identity and sexual orientation categories by respondent gender

Gender	Male (n=10, %)	Female (n=88, %)	Fisher’s exact test (p)
Heterosexual Woman	10(100%)	85(96.6%)	1.000
Heterosexual Man	2(20.0%)	44(50.0%)	0.098
Lesbian Woman	7(70.0%)	73(83.0%)	0.385
Gay Man	4(40.0%)	46(52.3%)	0.520
Bisexual Woman	7(70.0%)	72(81.8%)	0.402
Bisexual Man	3(30.0%)	43(48.9%)	0.327
Transgender Woman	7(70.0%)	52(59.0%)	0.735
Transgender Man	5(50.0%)	51(58.0%)	0.741
Questioning	4(40.0%)	53(60.2%)	0.312

Note: Positive evaluations include “Favorable” and “Very favorable,” corresponding to 3 and 4 on the 4-point scale. Gender differences for each attribute were tested using Fisher’s exact test. Statistical significance was set at p<0.05.

## Supplementary Material

PDF-Japanese
