# Supplementary material for: Acceptance of midwifery qualification acquisition by gender identity and sexual orientation: a survey of nursing students
Source: Fujita Med J. 2026 Feb 28;12(2):188–91. doi: 10.20407/fmj.2025-024 (PMC13129705; doi:10.20407/fmj.2025-024)
Supplement: Supplementary file 1 — PDF-Japanese [file fmj-12-188_s1.pdf]

Abstract
【背景】
多様な性の尊重が求められる中、日本では助産師資格の取得が法的に女性に限定され、出生時の性が男性である者には資格取得が認められていない。一方で、国際的には性別を問わず取得可能な国もあり、制度や社会的受容の在り方が問われている。
【目的】
看護学生の性自認・性的指向別の助産師資格取得に対する受容意識を明らかにする。
【方法】
2024 年 4 月～5 月、A 大学看護学科の 1~4 年生 98 名（男性 10 名、女性 88 名）を対象に、9 つの性自認・性的指向属性に対する助産師資格取得の受容度を 4 件法で評価する無記名オンライン調査を実施した。分析は、記述統計、クロス集計、χ^2検定を用いた。
【結果】
肯定的評価は「女性(異性愛)」が 96.9%と最も高く、「男性(異性愛)」「男性(バイセクシュアル)」が 46.9%と最も低かった。性別間で統計的に有意差は認められなかったが、一部の属性では実質的な男女差がみられた。
【結論】
助産師資格取得に対する受容は属性によって異なり、特に性役割や文化的イメージが影響している可能性がある。今後は、性別に関わらず誰もが助産師として活躍できる制度の見直しと多様性理解を促進する教育の必要性が示唆された。

キーワード：
助産師資格（Midwifery license）
性別による制限（Sex-based restriction）
性自認（Gender identity）
看護学生（Nursing students）
社会的受容（Social acceptance）

はじめに

近年、LGBTQ+を含む多様な性の在り方に関する議論や法整備は世界的に進展しており、日本でも LGBTQ+当事者の割合は約 9.7%を占めると報告されており[1]、2023 年には性別変更要件に関する最高裁判所の違憲判断が示されるなど[2]、多様な性を前提とした制度整備の必要性が高まっている。

日本では、看護師資格は性別にかかわらず取得可能である一方、助産師資格は保健師助産師看護師法により女性に限定されており[3]、出生時の性が女性でない者は資格取得が認められていない。これに対し、アメリカやイギリスなどの多くの国では、性自認や性的指向にかかわらず助産師資格を取得できる制度が整備されている[4]。

少子化や不妊治療、高齢出産の増加などを背景に、助産師の役割は一層重要となっており、多様な人材確保が喫緊の課題である。しかし、日本では、制度的制限や性別役割に対する社会的偏見が、助産師を志す人々にとって心理的・構造的障壁となっている可能性がある。海外では、男性やトランスジェンダーの助産師が就業しているが、教育や実践の場で孤立感や制度的困難に直面する事例が報告されており[5]、形式的な資格取得の可否にとどまらず、社会的受容や支援体制の整備が求められている。

フィリピンの看護学生を対象とした横断調査では、LGBT に対して肯定的な態度を示す学生が多数を占める一方で、知識水準は中程度にとどまり、両者の間に有意な相関は認められなかったとされる[6]。しかし、助産師資格取得に関する受容意識に焦点を当てた研究は、国内外を問わず極めて限られている。

本研究は、将来の医療提供者である看護学生を対象に、性自認および性的指向の異なる 9 つの属性に対する助産師資格取得の受容意識を明らかにし、今後の教育プログラムや制度改善に向けた基礎資料を提供することを目的とする。

方法

研究デザイン

横断的記述調査

調査対象者と調査方法

A 大学看護学科に在籍する 1~4 年生を対象に、2024 年 4 月〜5 月にかけて、Google フォームを用いた無記名式オンライン調査を実施した。調査協力は学内メールおよび対面での説明により依頼した。除外基準として、比較が困難であった 2020 年度入学者（1 名）は除外した。

調査項目

調査項目は以下の 3 つとした。①性別、②入学年度、③異性愛者を含む 9 つの性自認および性的指向属性に対する助産師資格取得の受容度（4 件法）。回答者の負担軽減のため、研究目的に沿った必要最小限の項目に限定した。本調査では年齢や助産師協会の役割認識も併せて尋ねたが、本稿では主要目的に関連する項目のみ分析・報告した。9 つの属性は、性自認および性的指向に関する先行研究[1]を基に選定し、さらに Human Rights Campaign[7] および GLSEN[8] の定義も参照した。

-女性（異性愛）

-男性（異性愛）
-女性（同性愛：レズビアン）
-男性（同性愛：ゲイ）
-女性（バイセクシャル）
-男性（バイセクシャル）
-トランス女性（身体の性は男性、性自認は女性）
-トランス男性（身体の性は女性、性自認は男性）
-クエスチョニング（自身の性自認や性的指向が不明）

分析方法

受容度は 4 件法（1＝非常に好ましくない、4＝非常に好ましい）で数値化し、各属性の評価について件数、割合、平均 ± 標準偏差を算出した。評価分布は棒グラフで可視化した。さらに、スコア 3 または 4 を「肯定群」、スコア 1 または 2 を「否定群」として二分化し、それぞれの割合を算出した。性別および入学年度との関連性はクロス集計を行い、χ^2検定または Fisher の正確確率検定で検討した。有意水準は両側検定で $p < 0.05$ とした。データ分析には SPSS Statistics（version 27, IBM Corp.）を使用した。

倫理的配慮

藤田医科大学医学研究倫理委員会の承認後、実施した（HM24-270）。

結果

回答者の背景

看護学科の学生 562 名に調査を依頼し、108 名から回答を得た（回収率 19.2%）。研究参加に不同意の 9 名および比較が困難であった 2020 年度入学者 1 名を除外し、最終的に 98 名（男性 10 名、女性 88 名）を分析対象とした（有効回答率 17.4%）。

各性自認・性的指向に対する助産師資格取得の評価分布（表 1 図 1）

最も肯定的評価が高かったのは「女性（異性愛）」96.9%で、次いで「女性（同性愛）」81.6%、「女性（バイセクシャル）」80.6%が続いた。最も低かったのは「男性（異性愛）」および「男性（バイセクシャル）」で、いずれも 46.9%であった。一方、その他の属性はいずれも肯定的評価が 50.0%以上であり、「トランス女性」60.2%、「クエスチョニング」58.2%、「トランス男性」57.1%、「男性（同性愛）」51.0%であった。全体として、性自認が女性の属性では肯定的評価が高く、性自認が男性の属性では低い傾向が認められた。

（表 1・2、図 1 挿入）

回答者の性別との関連（表 2）

Fisher の正確確率検定では、性別間に統計的有意差は認められなかった（$p > 0.05$）。一方で、

肯定的評価の割合は、「トランス女性」は男性 70.0%、女性 59.0%（$p = 0.735$）、「男性（異性愛）」は男性 20.0%、女性 50.0%（$p = 0.098$）と、統計的には有意ではないものの 10-30 ポイントの差がみられた。

入学年度との関連（Supplementary Material 1）

　入学年度別の内訳は、2021 年度 24 名（17.6%）、2022 年度 25 名（19.1%）、2023 年度 26 名（17.3%）、2024 年度 23 名（15.9%）であった。補足的に評価を検討した結果、2022 年度および 2023 年度の学生で肯定的評価が相対的に高かった。「男性（異性愛）」および「男性（バイセクシャル）」では有意差が認められた（$p < 0.05$）。

考察

　本研究は看護学生を対象に、多様な性自認・性的指向に基づく助産師資格取得に対する受容意識を明らかにした点に新規性がある。

　調査の結果、肯定的評価が 50%を下回ったのは「男性（異性愛）」および「男性（バイセクシュアル）」の 2 属性のみであり、他の 7 属性はいずれも過半数の肯定的評価が得られた。これは、評価が単に身体的性別（生物学的性）に基づいているのではなく、助産師に対する文化的イメージや性別的役割への期待が影響している可能性を示唆する。また、「トランス女性」や「異性愛（男性）」に対する評価において、回答者の性別による肯定率の差が 10〜30 ポイントみられ、同性の属性に対する心理的距離感やジェンダー役割に対する意識の違いが影響している可能性が考えられる。今後は、性別ごとの評価傾向を大規模な量的研究により精緻に検証するとともに、質的研究を通じてその背景を深掘りすることが望まれる。

　補足的に検討した入学年度別の分析では、「男性（異性愛）」および「男性（バイセクシュアル）」の 2 属性において有意差が認められ、2022 年度および 2023 年度の入学者で肯定的評価が高かった。ただし回答者数は限られているため、選択・検出バイアスの影響を受けた可能性があり、教育環境や社会背景を直接の要因とする根拠は乏しい。一般化は困難であり、今後は大規模調査が必要である。

　日本における助産師の歴史は江戸時代に遡り、明治期に制度化された[9]。先行研究では、女性は出産時に「女性に助けてもらいたい」という文化的期待が強く、助産師としての女性を好む傾向があることが示されている[10]。このような背景から助産師は「出産を支える母性的な女性」といった認識が依然として残っていると考えられる。また、日本では男性は助産師資格を取得できないため[3]、この制度的制限が助産師像を女性限定の職業として固定化し、社会的認識に影響を与えている可能性がある。こうした固定的な価値観や制度的制限は、無意識のうちに職業選択や評価へ影響を及ぼす可能性がある。実際、トランスジェンダーおよびジェンダー・エクスパンシブの医療従事者に関する研究では、トランスフォビアによる心理的苦痛や性別二元論、制度的障壁による孤立感が報告されており、環境整備の重要性が指摘されている[11]。

　本研究では、文化的助産師像と親和性の高い属性は肯定的に評価される一方、そうでない属性は制度的・文化的背景の影響で抑制される傾向がみられた。助産師像の形成には、性別だけでなく社会的・経済的背景や個人経験も影響するとされる[12]。看護学生は性の多様性に理解を示しや

すい集団と考えられるが、医療者に無意識の性的マイノリティへのバイアスがあること[13]や、看護学生の中立的な態度[14]、知識の不足[15]も報告されており、この点も考慮が必要である。なお、助産師資格の取得に対する受容意識を調査したが、資格取得と実際に助産師として活動することには異なる社会的受容が存在する可能性がある。今後は、資格取得に対する受容と実際のサービス提供の受容の違いを比較検討することが望まれる。

今回得られた知見は、教育課程において多様な助産師像を提示し、ジェンダーや性的指向に起因する偏見を低減する教材開発や研修に活用できる。加えて、国際的に性別要件を撤廃する国が複数存在する現状と整合し、助産師資格の性別要件の見直しや多様な人材が参画できる制度設計に向けた基礎資料として重要な示唆を与える。

研究限界

第一に、サンプル数が 98 名と少なく、属性ごとの比較において統計的検出力が十分でなかった可能性がある。第二に、調査対象が看護学科の学生に限定されており、一般市民の意識とは異なる可能性がある。第三に、インターネット調査で実施されたため、社会的望ましさバイアスや回答者特性の偏りが生じた可能性がある。これらを踏まえ、今後は多様な対象者を含めた大規模調査による検証が求められる。

結論

本研究は、助産師資格取得に対する社会的受容を明らかにすることを目的に、性自認・性的指向の異なる 9 属性に対する看護学生の受容意識を調査した。その結果、「女性属性」に対する肯定的評価が高く、「男性属性」では低い傾向がみられた。一方で、異性愛以外の性的指向や出生時の性と異なる性自認をもつ属性にも肯定的評価が得られ、社会的受容の広がりが示唆された。今後は、異なる背景における評価傾向を比較し、教育プログラムや制度改善に資する知見を蓄積することが求められる。

利益相反

本研究における利益相反はない。

謝辞

この研究に参加いただいたすべての方に感謝申し上げます。

引用文献

1. Dentsu Group Inc . Dentsugurupu lgbtq chosa 2023 o jisshi (The 6th LGBT Survey); 2023 (in Japanese). <https://www.group.dentsu.com/jp/news/release/pdf-cms/2023046-1019.pdf>(Accessed May 13, 2025)
2. Japan Lawyers Association for Freedom. Saikosai niyoru seibetsu henko no shujutsu

yoken “iken” handan o uke, seiteki mainoritei no genjo ni mukiai, kofuku tsuikyuken o fumaeta shiho to rippo no arikata o motomeru seimei(Supreme Court rules sterilization requirement unconstitutional); 2023 (in Japanese). <https://www.jlaf.jp/04seimei/2023/1027_1647.html> (Accessed May 13, 2025)

3. Ministry of Health, Labour and Welfare. Hokenshi josanshi kangoshi ho. dai 3 jo (Act on Public Health Nurses, Midwives and Nurses, Article 3); 2023 (in Japanese). <https://elaws.e-gov.go.jp/document?lawid=323AC0000000203> (Accessed May 13, 2025)
4. Sannomiya M, Sasagawa E, Hikita N, Yonezawa K, Haruna M. The Proportions, Regulations, and Training Plans of Male Midwives Worldwide: A Descriptive Study of 77 Countries. Int J Childbirth 2019; 9 5-18.
5. Kantrowitz-Gordon I, Adriane Ellis S, McFarlane A. Men in Midwifery: A National Survey. J Midwifery Womens Health 2014;59:516-22.
6. Oducado RMF. Knowledge and attitude towards lesbian, gay, bisexual, and transgender healthcare concerns: A cross-sectional survey among undergraduate nursing students in a Philippine state university. Belitung Nurs J. 2023; 9: 498-504.
7. Human Rights Campaign. Glossary of terms;2024. <https://www.hrc.org/resources/glossary-of-terms> (Accessed July 14, 2025)
8. Gay, Lesbian & Straight Education Network (GLSEN). Terms and concepts;2020. <https://www.glsen.org/sites/default/files/2020-04/GLSEN%20Terms%20and%20Concepts%20Thematic.pdf> (Accessed July 14, 2025)
9. Japan Midwives Association. Josanshi toha horitsu to teigi(History of Midwifery in Japan) (in Japanese). <https://www.midwife.or.jp/midwife/about.html> (Accessed May 13, 2025)
10. Savella M, Savella GA. The context of male midwives among rural communities. ASEAN Journal of Community Engagement 2022;6:1–21.
11. Westafer LM, Freiermuth CE, Lall MD, Muder SJ, Ragone EL, Jarman AF. Experiences of transgender and gender expansive physicians. JAMA Netw Open 2022;5:e2219791.
12. Monne R, Iddrisu M, Kyilleh JM, Ibrahim MM, Nashiru AR, Wuni A, Yariga FY, Teye-Djangmah D, Abdulai AM. Male midwifery practice and acceptability: attitudes, beliefs, and associated factors among expectant mothers in Savelugu Municipal Hospital, Northern Region of Ghana. Pan Afr Med J 2024;47:199.
13. Sabin JA, Riskind RG, Nosek BA. Health Care Providers' Implicit and Explicit Attitudes Toward Lesbian Women and Gay Men. Am J Public Health. 2015;105:1831-41.
14. Özdemir RC, Erenoğlu R. Attitudes of nursing students towards LGBT individuals and the affecting factors. Perspect Psychiatr Care. 2022;58:239-247.
15. Wang YC, Miao NF, You MH. Attitudes toward, knowledge of, and beliefs regarding providing care to LGBT patients among student nurses, nurses, and nursing educators: A cross-sectional

survey. Nurse Educ Today. 2022;116:105472.

表 1.各性自認・性的指向属性に対する助産師資格取得の評価分布（4 件法）および記述統計

Note: 回答は 4 件法（1＝非常に好ましくない〜4＝非常に好ましい）で実施された。各属性ごとに、各選択肢の回答割合（%）、平均値および標準偏差（Mean ± SD）を示している。また、1・2 を「否定」、3・4 を「肯定」として 2 値化し、それぞれの回答数（n）と割合（%）も併せて示した。

表 2. 性別および性自認・性的指向属性による助産師資格の取得の受容評価（肯定的評価）

図 1. 各性自認・性的指向属性に対する助産師資格取得の評価分布（4 件法）

Note: 肯定的評価（「好ましい」「非常に好ましい」）の合計が高い順に表示している。

補足資料 1. 入学年度および性自認・性的指向属性による助産師資格取得の受容評価

Note: 肯定的な回答には「好ましい」および「非常に好ましい」の 4 段階評価を含む。性別および入学年度ごとの差は χ^2検定を用いて検定され、有意水準は $p < .05$ に設定した。
