# Supplementary material for: Acceptance of midwifery qualification acquisition by gender identity and sexual orientation: a survey of nursing students
Source: Fujita Med J. 2026 Feb 28;12(2):188–91. doi: 10.20407/fmj.2025-024 (PMC13129705; doi:10.20407/fmj.2025-024)
Supplement: Supplementary file 2 — Supplementary Material [file fmj-12-188_s2.pdf]

Supplementary Material 1. Proportions of positive evaluations across gender identity and sexual orientation categories by year of enrollment

Gender	2021 [n=24, %]	2022 [n=25, %]	2023 [n=26, %]	2024 [n=23, %]	χ^2 test (p)
Heterosexual Woman	24(100.0%)	24(96.0%)	26(100.0%)	21(91.3%)	0.249
Heterosexual Man	6(25.0%)	14(56.0%)	17(65.2%)	9(39.1%)	0.023
Lesbian Woman	20(83.3%)	20(80.0%)	24(92.3%)	16(69.6%)	0.231
Gay Man	8(33.3%)	14(56.0%)	18(69.2%)	10(43.5%)	0.065
Bisexual Woman	18(75.0%)	21(84.0%)	24(92.3%)	16(69.6%)	0.192
Bisexual Man	7(29.1%)	14(56.0%)	17(65.4%)	8(34.8%)	0.032
Transgender Woman	11(45.8%)	17(68.0%)	18(69.2%)	13(56.5%)	0.294
Transgender Man	12(50.0%)	16(64.0%)	19(73.1%)	9(39.1%)	0.081
Questioning	12(50.0%)	17(68.0%)	19(73.1%)	9(39.1%)	0.059

Note: Positive evaluations include “Favorable” and “Very favorable,” corresponding to 3 and 4 on the 4-point scale. Differences among admission years were analyzed using the χ^2 test, with statistical significance set at $p < 0.05$.
